# Restored oligodendrogenesis by fibroblast growth factor 17: molecular mechanism for rejuvenating ageing-related memory deficit

**DOI:** 10.1038/s41392-022-01092-x

**Published:** 2022-07-16

**Authors:** Xiao-Yi Xiong, Alexey Semyanov, Yong Tang

**Affiliations:** 1 School of Acupuncture and Tuina, Chengdu University of Traditional Chinese Medicine; Acupuncture & Chronobiology Key Laboratory of Sichuan Province, 610075 Chengdu, China; 2 Department of Molecular Neurobiology, Shemyakin-Ovchinnikov Institute of Bioorganic Chemistry, Russian Academy of Sciences, Miklukho-Maklaya Street 16/10, Moscow, 117997 Russia; 3 Sechenov First Moscow State Medical University, Bolshaya Pirogovskaya Str 19с1, Moscow, 119146 Russia; 4 International Collaborative Centre on Big Science Plan for Purinergic Signalling, Chengdu University of Traditional Chinese Medicine; School of Health and Rehabilitation, Chengdu University of Traditional Chinese Medicine, 610075 Chengdu, China

**Keywords:** Molecular neuroscience, Neurodevelopmental disorders

In a recent article published in *Nature*,^1^ Iram et al. are the first to identify fibroblast growth factor 17 (Fgf17) from the young mice cerebrospinal fluid (YM-CSF) as a key molecule to improve cognitive functions in aged mice by driving the proliferation and differentiation of oligodendrocyte progenitor cells (OPCs), indicating that effective remyelination induced by young CSF factors may provide a novel and promising therapeutic strategy to rejuvenate the ageing brain, which again confirms that factors present in the young tissue can serve as therapies in ameliorating ageing-related symptoms.

People have been searching for the secret of longevity since ancient times, but no breakthrough has been made to date. However, leaving aside the question of whether longevity is feasible, there is an urgent need to resolve the issue of ageing-related health deterioration. For example, cognitive decline remains an unsolved issue for the elderly. The senescence of neurons has long been considered as a major cellular mechanism of such decline.^2^ However, the recently developed concept of brain active milieu suggests that the brain functions are determined by orchestrated interactions among neurons, glia, non-neuronal cells, and non-cellular elements.^3^ Thus, senescence of all elements of the active milieu can play a role in brain ageing. For example, we recently showed that astrocytic dystrophy parallels the impairment of synaptic plasticity in old brains.^4^ In addition, oligodendrocytes and they produced myelin sheaths have also been shown to play crucial roles in learning and memory,^2^ because a long-range signaling in the brain that relies on myelin functions and mature oligodendrocytes are important for the maintenance of axonal health and for regulation of neuronal function. Therefore, myelin degeneration and diminished myelin renewal contribute to ageing-related deficits in memory.^5^ An interesting finding showed that myelination is very intense in young mice and is greatly inhibited in aged mice, coinciding with spatial memory deficits.^5^ Unfortunately, the detailed reasons of the marked differences in myelin regeneration between young and old remains unclear. As we know, myelin remodeling in brain is primarily orchestrated by mature oligodendrocytes, which are derived from OPCs’ differentiation. Therefore, maintaining the dynamic balance between OPCs and oligodendrocytes during aging to promote effective myelin remodeling could be beneficial for improving ageing-related symptoms.

In this study, Iram et al. first tested whether administration of young CSF to aged mice via intracerebroventricular (ICV) would effectively rejuvenate the brain. When age-related cognitive decline (e.g., remote memory recall) was reversed by YM-CSF, they investigated the cellular and molecular changes in hippocampus with bulk RNA sequencing (RNA-seq). They found that highly upregulation of transcription factors driven by YM-CSF were involved in promoting oligodendrocyte differentiation and major myelin protein components. Given that the OPCs are the initiating myelination process, their growth was assessed in the aged hippocampus and in primary OPC cultures. The results showed that YM-CSF enhances OPC proliferation and differentiation both in in vivo and in vitro. These results suggest that young CSF contains rejuvenating factors that promote cell growth for the oligodendrocyte lineage or neutralize inhibitory factors.

Next, to gain a deeper mechanistic insight into the cell growth for OPCs induced by YM-CSF, nascent mRNAs from cultured OPCs 1 or 6 h after exposure to young human CSF (YH-CSF) were metabolically labeled with 4-thiouridine (s4U) and then detected by thiol (SH)-linked alkylation for the metabolic sequencing of RNA (SLAMseq). The results of this experiment showed that many of the differentially expressed genes peaking at 6 h after YH-CSF exposure are known as serum response factor (SRF, a transcription factor driving actin cytoskeleton rearrangement) target genes. The most strongly upregulated genes were enriched for “target genes of SRF”. The following experiments demonstrated that the suppression of the OPC proliferation and differentiation in the aged brains was caused by the depressed SRF signaling pathway in OPCs, which was reactivated in vivo by young CSF treatment. Therefore, these experiments indicate that SRF signaling in OPCs is inhibited with ageing and may serve as a therapeutic target against the ageing-related deficits.

Because the source and quantity of CSF may be the most critical factors restricting its widespread application in clinical practice, revealing the active components of CSF can address this issue. Then, based on the observation of SRF signaling pathway, two published CSF proteomic datasets were cross-referenced with the predicted SRF targets, which generating a list of 35 potential SRF inducers. Among them, Fgf17 gained the favor of researchers because it is a brain-enriched protein, and its levels decrease with age in human plasma, CSF and mouse neurons. It also induced the strongest dose-dependent activation of SRF. In addition, Iram et al. found that Fgf17 activates SRF signaling through actin modulation by enhancing or inhibiting actin polymerization using jasplakinolide and latrunculin A, respectively. In vitro experiment showed that Fgf17 induces OPC proliferation and differentiation when primary rat OPCs exposure to Fgf17. To further confirm these effects observed in in vitro, the aged mice received 1 week of infusion with Fgf17 showed markedly increased OPC proliferation in the aged hippocampus and improved long-term memory performance. In contrast, blocking Fgf17 in young mice showed impaired performance and impaired neuronal plasticity, suggesting that Fgf17 is necessary for normal memory function. These results strongly demonstrate that Fgf17 is sufficient to activate OPCs in the hippocampus and consolidate memory in aged mice, which was comparable to the effects of young CSF.

**Fig. 1:**
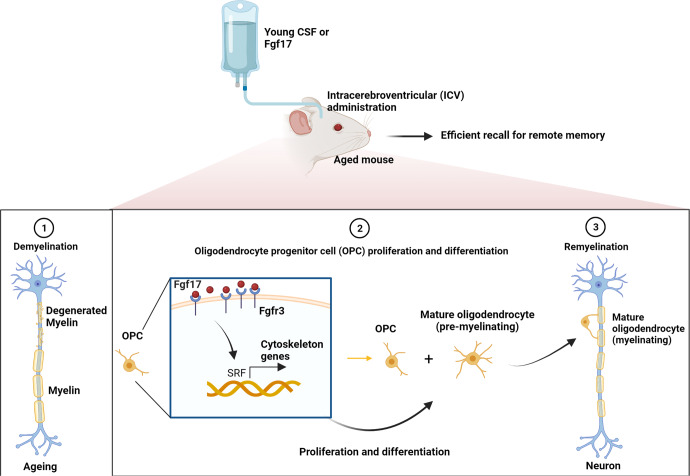
Mechanism of Young CSF or Fgf17 infusion mediated oligodendrogenesis. OPC proliferation and differentiation (termed oligodendrogenesis) were inhibited with age. Young CSF or the brain-specific growth factor Fgf17 infusion boosts hippocampal oligodendrogenesis via reactivating SRF signaling pathway and improved the long-term memory recall of aged mice

Iram et al. have provided solid evidence that young CSF is capable of restoring memory in aged mice via Fgf17 at the OPCs (Fig. 1). Although the possibility that CSF proteins directly affect mature oligodendrocyte function cannot be excluded by this study, the finding of the rejuvenating power of young CSF on restoring oligodendrogenesis in aged mice may greatly inspire researchers to explore whether the young rejuvenating factors are beneficial to the demyelination-related diseases. Taken together, this intriguing work makes a great contribution to the field of remyelination and provides potential therapeutic targets for improving memory in dementia and neurodegenerative disorders.
